# Papillomavirus binding factor (PBF) is an intrinsically disordered protein with potential participation in osteosarcoma genesis, *in silico* evidence

**DOI:** 10.1186/1742-4682-11-51

**Published:** 2014-12-03

**Authors:** Paola Castillo, Abraham F Cetina, Alfonso Méndez-Tenorio, Lennane Michel Espinoza-Fonseca, Blanca L Barrón

**Affiliations:** Department of Microbiology, Escuela Nacional de Ciencias Biológicas, Instituto Politécnico Nacional, Carpio y Plan de Ayala S/N, Casco de Santo Tomás, México, DF 11340 México; Department of Biochemistry, Escuela Nacional de Ciencias Biológicas, Instituto Politécnico Nacional, Carpio y Plan de Ayala S/N, Casco de Santo Tomás, México, DF 11340 México; Department of Biochemistry, Molecular Biology and Biophysics, University of Minnesota, Minneapolis, MN 55455 USA

**Keywords:** Zinc finger protein 395, Osteosarcoma, Intrinsically disordered proteins (IDP), Transcription factor

## Abstract

**Background:**

Papillomavirus binding factor (PBF) or zinc finger protein 395 is a transcription factor associated to a poor prognosis in patients with osteosarcoma, an aggressive bone cancer that predominantly affects adolescents. To investigate the role of the PBF protein in the osteosarcoma genesis, in this paper we present the bioinformatics analysis of physicochemical properties of PBF and its probable interactions with several key cellular targets.

**Results:**

The physicochemical characteristics determined to PBF, disorder-promoting amino acids, flexibility, hydrophobicity, prediction of secondary and tertiary structures and probability to be crystallized, supported that this protein can be considered as an intrinsically disordered protein (IDP), with a zinc finger-like domain. The *in silico* analysis to find out PBF interactions with cellular factors, confirmed the experimentally demonstrated interaction of PBF with two key cellular proteins involved in regulation of cellular apoptosis, 14-3-3β and Scythe/BAT3 proteins. Furthermore, other interactions were found with proteins like HDAC1 and TPR which are known to be deregulated in several cancers. Experimental confirmation of specific interactions will contribute to understand the osteosarcoma process and might lead to the identification of new targets for diagnosis and treatments.

**Conclusions:**

According to the *in silico* PBF analyses, this protein can be considered as an IDP capable to bind several key cellular factors, and these interactions might play an important role in the osteosarcoma process.

## Background

Osteosarcoma is the most common type of bone cancer. It is a very aggressive cancer and is the sixth leading cancer in children under age 15, and more than 92% of biopsy specimens of osteosarcoma have shown a protein known as papillomavirus binding factor (PBF) highly expressed in the cellular nucleus [1]. PBF, also known as zinc finger protein 395, is a 513 amino acids cellular transcriptional factor that regulates the activity of the human papillomavirus late promoter, recognizing the sequence CCGG of the E2 binding site [2].

Clinical studies have revealed that PBF-positive osteosarcoma patients were associated with a significantly poorer prognosis than those with negative PBF expression. In addition, overexpression of PBF has been reported in many cases of bone and soft tissue sarcoma and epithelial carcinomas [1]. All these evidences have suggested that PBF has a central role in osteosarcoma genesis, and therefore PBF might be used as a potential therapeutic target for anti-cancer drugs. Even more, PBF has been identified as a cytotoxic T lymphocytes-defined osteosarcoma antigen in the context of human leukocyte antigen (HLA)-B*5502 [3, 4]. Besides that, in 2008 Tsukahara *et al.,* [1] developed a synthetic antigenic peptide from PBF capable to induce cytotoxic T lymphocytes from an HLA-A24-positive patient which specifically killed an osteosarcoma cell line expressing PBF and HLA-A24.

PBF has been studied in several cancer cells, but its function in normal cells remains to be elucidated. PBF has shown a nuclear-cytoplasmic localization, suggesting that its transcription role is related to its cellular localization. Besides that, several interactions with other cellular molecules have been reported. PBF is capable to bind to 14-3-3β protein (Tyrosine 3-monooxygenase/tryptophan 5-monooxygenase activation protein, beta); member of the 14-3-3 family proteins which play a role in cell cycle regulation, apoptosis and malignant transformation. It has been proposed that 14-3-3β binding to PBF might inhibit the cell growth [5]. PBF overexpression has demonstrated to induce apoptosis in cancer cells; and PBF has also been found interacting with Scythe/BAT3 (Large proline-rich protein), an anti-apoptotic protein with an important role in cell proliferation. Scythe/BAT3 and PBF have been co-localized in the nuclei of osteosarcoma cells, and probably this interaction is responsible of apoptosis inhibition [6]. All these studies have suggested that PBF might have an important role in the osteosarcoma genesis. For that reason, we carried out an *in silico* analysis of PBF, 1) first of all to determine if PBF might be considered as an intrinsically disordered protein (IDP). The IDPs are a new protein group that lack of stable tertiary and/or secondary structures in physiological or *in vitro* conditions, and it is known that IDPs are abundant in eukaryotic cells. In fact, it has been estimated that approximately 25% of mammalian proteins are intrinsically disordered and about 75% of proteins involved in signaling and regulation are partially or fully unfolded [7–15]. In order to engage in intermolecular interactions with various targets in the cell, IDPs use short sequential recognition elements, usually known as primary contact sites, preformed structural elements or molecular recognition elements/features (MorEs/MorFs) [16–19]. 2) Secondly, if PBF is an IDP it probably might be able to interact with several cellular factors affecting signaling pathways which could activate the osteocarcinogenesis process. For that, we also used several bioinformatics tools to identify probable cellular factors capable of interacting with PBF and participate in the genesis of the osteosarcoma.

## Results and discussion

To investigate the role of the PBF protein in the osteosarcoma carcinogenesis process, we analyzed if the PBF protein could be considered as an intrinsically disordered protein (IDP).

### Prediction of structural disorder in PBF and nuclear localization

#### Disorder-promoting amino acids and zinc finger

**Figure 1:**
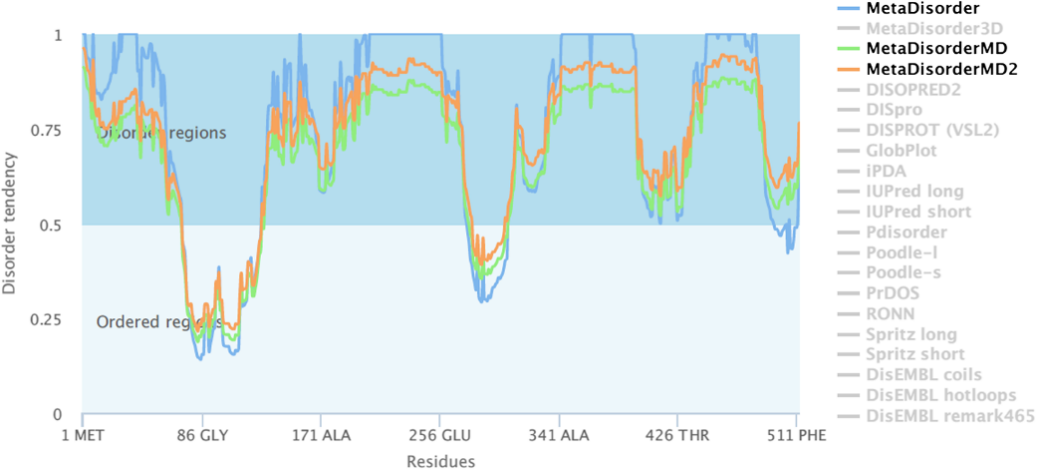
**Disorder analysis of PBF using the MetaDisorder server.** The graph shows the disorder tendency of PBF amino acids sequence, analyzed by three versions of MetaDisorder: blue, green and orange lines. All three, produced similar results, and PBF can be considered as a protein with highly tendency to be disordered (values above 0.5), and three clearly disordered regions were located at amino acids position 1–72, 131–216 and 304–513.

The analysis of the amino acids sequence of PBF showed that about 62% of PBF is composed by the so-called disorder-promoting amino acids [15, 20, 21]. In particular, strong disorder-promoting amino acids; proline, serine and glutamic acid, together accounted for almost 29% of the amino acids content of PBF (Table 1). Disorder-promoting amino acids, like arginine, glycine, serine, glutamic acid, lysine and proline, prevent folding into discrete structural states. The high proportion of disorder-promoting amino acids in the sequence of PBF suggests that this protein is likely to be partly or fully disordered. To determine disordered regions inside PBF, we used three servers, Intfold, disEMBL and MetaDisorder, obtaining similar results. The amino acids residues located at positions 1 to 72, 131–216, and 304–513, showed high disorder level. Even more, these servers coincided in the presence of two ordered regions; one located at amino acids position 86–128, and other, at the amino acids 279–302 (Figure 1 and Table 2). This last ordered region corresponded to a putative zinc finger motif which might be stabilized by the beta sheets and alpha helix located in it as it was described for the human cellular protein ZNF593 (Zinc finger protein 593), which is a negative modulator of the DNA-binding activity of the Oct-2 transcription factor [22]. The PBF putative zinc finger coincided with the proposal presented by Boeckle *et al*., in 2002 [2], based on the amino acids sequence which revealed the presence of pairs of cysteines (282 and 287 amino acids position) and histidines (300 and 305 amino acids position) spaced by 12 amino acids; conforming a classical zinc finger of TFIIIA type.

**Table 1:** **Disorder promoting amino acids of PFB**

Disorder-promoting amino acids				Order-promoting amino acids		Other	
Polar		Smalls		Hydrophobic or bulky		Amino acids	
Ser	**11.90%**	Ala	10.70%	Val	6.20%	Leu	7.40%
Pro	**11.30%**	Gly	6.80%	Phe	2.30%	Thr	4.50%
Glu	**5.80%**			Trp	2.10%	Asp	3.90%
Gln	5.70%			Tyr	1.90%	Cys	2.70%
Lys	5.30%			Ile	1.80%	His	2.50%
Arg	4.50%			Met	1.80%	Asn	0.80
Total 62%				Total	16.10%	Total	21.9%

Analysis of PBF amino acids using the ProtParam server. The bold data indicates the strong disorder promoting amino acids found in PBF.

**Table 2:** **Anatomy and functions of PBF**

Domain^a^	Disordered or ordered structure^b^	Probable phosphorylation sites^c^	Probable binding sites to cellular factors^d^	Function
I (1–125)	Disordered region (1–72) Ordered region (86–128)	7 sites	1 site (1–22)	Unknown
II (126–215)	Disordered region (131–216)	8 sites	2 sites (166–182; 193–206)	Unknown
III (216–301)	Ordered region (279–302)	11 sites	1 site (277–304)	-Monopartite NLS (PAPRKRKNSVK, 267–277)^e^
				-Zinc finger domain (279–302)
				-Rich G-C DNA binding site of HPV type 8 [1]
IV (302–483)	Disordered region (304–513)	18 sites	3 sites (320–341; 359–372; 393–416)	-Binding to 14-3-3β (393–398; **444**–**453**). PBF must be phosphorylated at Ser 447, 449 and 451 [5].
				-Sequence recognized by cytotoxic T cells in the context HLA-A24 [2].

Parentheses indicate the amino acid residues position. Bold residues indicate the high affinity site. Identified in this paper using: ^a^Mammoth analysis; ^b^Intfold, disEMBL and Metadisorder analyses; ^c^NetPhos 2.0; ^d^ANCHOR; ^e^cNLS Mapper, NucPred, and PSORT II analyses.

#### Flexibility

**Figure 2:**
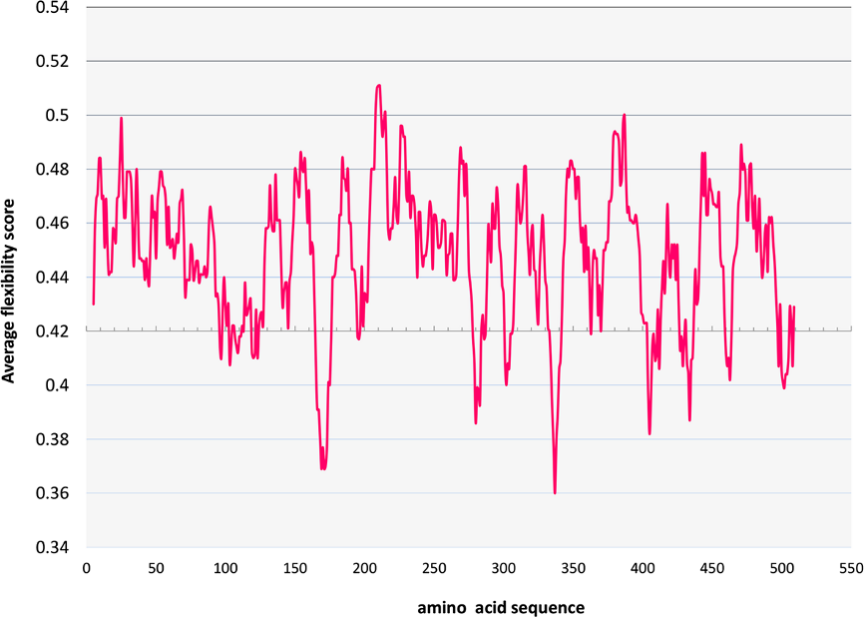
**PBF average flexibility determined using the EMBOSS server.** The graph shows the average flexibility score for PBF amino acids sequence. More than 90% of PBF amino acids were located in flexible regions, values above 0.42; indicating that PBF is a highly flexible protein.

Other important characteristic in the IDPs is the flexibility. It is known that high flexibility levels allow fast structural changes to the protein; but also, provide highly specific low affinity interactions. The flexibility analysis of PBF with the Expasy server using an average flexibility scale, showed many flexible regions along the sequence. These flexible regions were interrupted by short areas of less flexibility, as it is shown in Figure 2. Therefore PBF can be considered as a highly flexible protein. The flexibility property may contribute to PBF biological functions, allowing its interaction with different cellular targets, such as has been described for p53 (tumor suppressor protein). This important transcription factor is an IDP involved in DNA repair, cell progression, apoptosis induction, senescence and response to cellular stress. Even more, its C-terminal domain is very flexible and can adopt four different structures; short α-helix, β-strand and two different coils, these changes allow its binding to several partners [21, 23, 24].

#### Hydrophobicity

**Figure 3:**
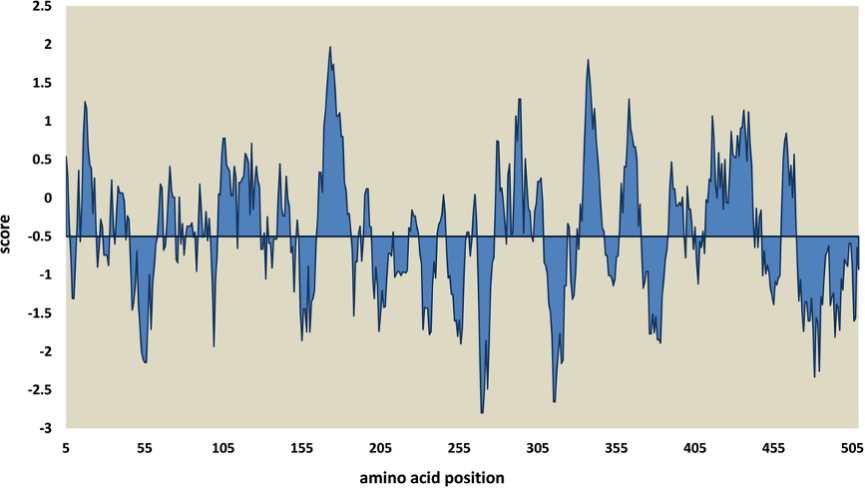
**PBF hydrophobicity determined using the EMBOSS server with the Kyte and Doolite scale.** Regions below −0.5 score are considered hydrophilic areas. The figure shows that approximately 50% of PBF amino acids were located at hydrophilic regions.

Amino acid residues analysis of PBF, showed a high content of polar amino acids (58.9%). According to the hydrophobicity results using Kyte and Doolitle scale (Figure 3) we found that approximately 50 percent of the PBF amino acid residues were located at hydrophilic regions. It is well known that a combination of low hydrophobicity (leading to low force for protein compaction) and high net charge (leading to strong electrostatic repulsion) are important requisites for the absence of a compact protein structure, which is seen in the IDPs [7, 21].

### Prediction of secondary and tertiary structure of PBF

It has been established that IDPs lack of that stable second and tertiary structure under physiological and *in vitro* conditions, due to its high flexibility and random coil conformation [20]. The ability of a protein to fold or not fold under physiological conditions depends of several factors; 1) the amino acids sequence, 2) the combination of low mean hydrophobicity, which leads to a low driving force for protein compaction, and 3) a high net charge, which generates a strong electrostatic repulsion [20, 21]. To determine if PBF could have a compact structure we analyzed its probability to be crystallized using XtalPred server, which generates a probability score for crystallization. The scale is 1 to 5; 1 is the score for proteins with high probability of being crystallized, while 5 is for proteins with very low probability. We compared the probability of PBF and the RUBISCO protein to be crystalized; obtaining a 5 score for PBF, meaning it is a very difficult task, in contrast the RUBISCO protein (PDBID:1UZH), showed a value of 3 (Data not shown).

**Figure 4:**
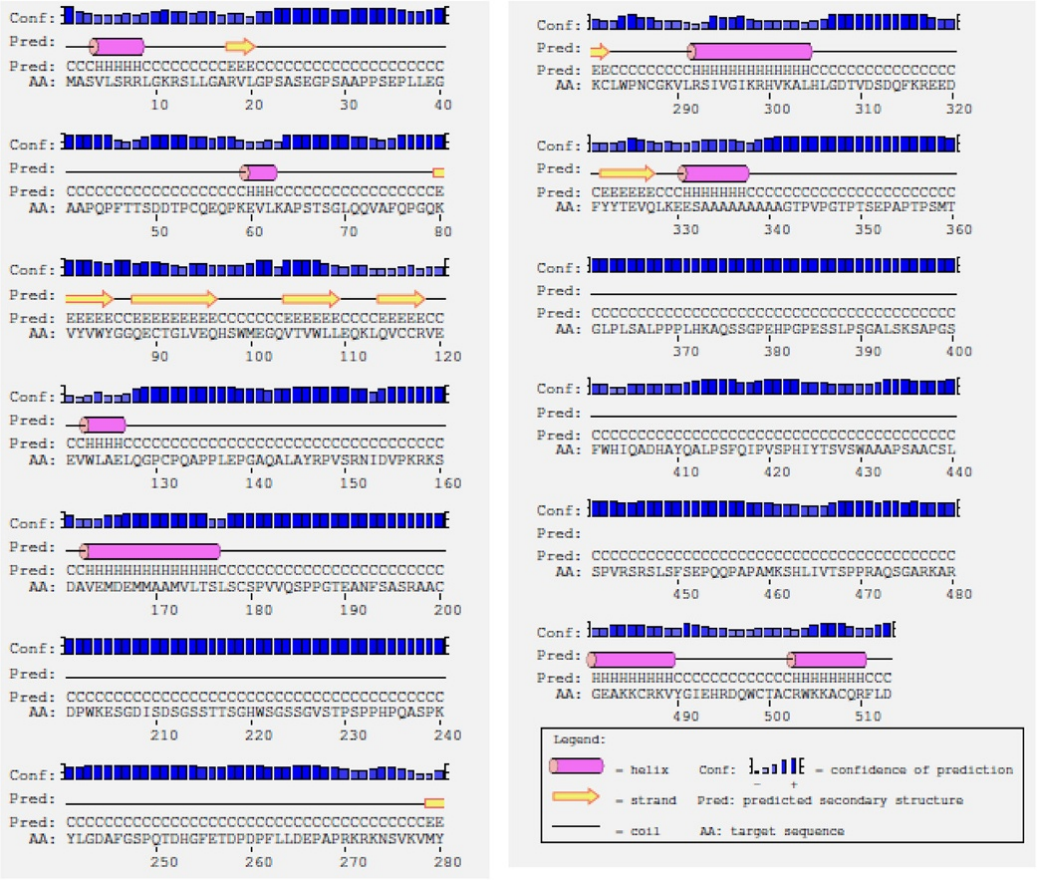
**PBF secondary structure predicted with the Psipred server.** The PBF secondary structure showed to be organized in long loops or coil regions interrupted with short beta sheets and alpha helices. The Psipred model showed; eight alpha helices (pink barrels) and seven beta sheets (yellow arrows); one alpha helix and one beta sheet were located among amino acid positions 216–301. Data in agreement with the putative zinc finger proposed in this region.

**Figure 5:**
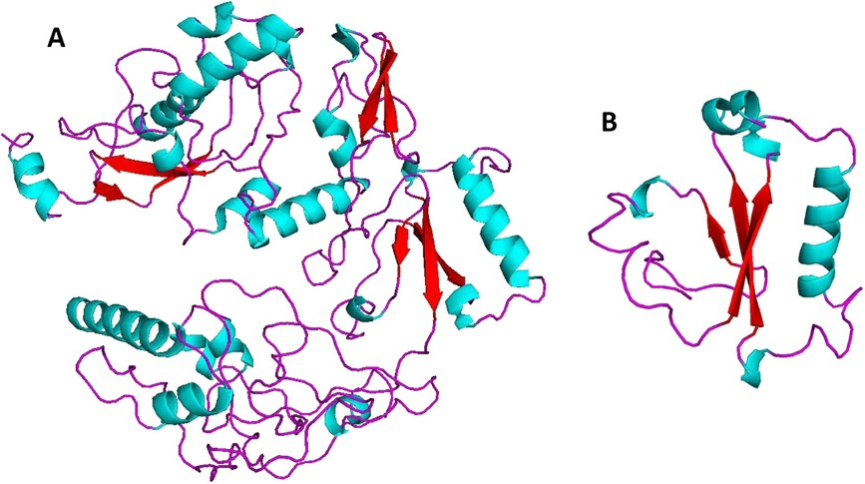
**PBF tertiary structure predicted with the Robbeta server. A)** PBF predicted 3D structure showing long coils in magenta, interrupted by short alpha helices in blue, and beta sheets in red. **B)** The 3D structure at amino acid residues 216–301, showing the putative zinc finger domain.

Due to the fact there is not any report about crystallization of PBF or PBF homologous, we modeled *in silico* the tertiary PBF protein structure using the Robbeta server. Initially five models were obtained and we chose the model with the best stereochemistry quality, 89.5% of the amino acid residues were located in favoured regions using Procheck (Data not shown). On the other hand, comparison of PBF secondary and tertiary structures showed in both cases an structure formed mainly by long coils or loop structures interrupted by short beta sheets and alpha helixes (Figures 4 and 5); and both coincided with the presence of a beta sheet and an alpha helix located among amino acids 216–301 (Figure 5B). Specifically the beta sheet is formed by the amino acids MYKC; while the alpha helix is formed by the amino acids LRSSIVGIKRHVKALH. These sequences were located in the putative zinc finger proposed by Boeckle *et al*., 2002 [2].

### Phosphorylation sites in PBF

**Figure 6:**
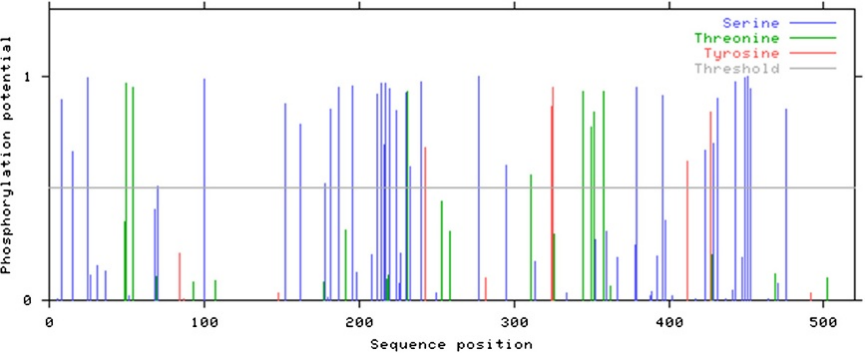
**PBF phosphorylation sites predicted using the NetPhos 2.0 Server.** PBF probable phosphorylation sites along the amino acids sequence are indicated in different colors, blue lines for serine phosphorylation, green lines for threonine; and red line for tyrosine. The probable phosphorylation sites detected are in agreement with the amino acids content of PBF, 11.9% serine, 4.5% threonine and 1.9% tyrosine.

It is known that protein phosphorylation is an important post-translational modification and a key regulatory step in different cellular processes [25]. Furthermore, phosphorylation sites are linked with disordered regions, allowing transient but specific interactions with different targets [25]. The phosphorylation analysis of PBF indicated several probable phosphorylation sites along the protein amino acids sequence. Among amino acids 304–513 there were 18 probable phosphorylation sites (Figure 6). Even more, it is known that in this region PBF binds to the 14-3-3β protein; but PBF must be phosphorylated at the serine amino acid residues 447, 449 and 451, and all of these phosphorylation sites were located in a disordered region (Table 2). The complex phosphorylated PBF-14-3-3β protein has been shown to be translocated into the cellular nucleus where it might inhibit cell apoptosis [5]. Other PBF potential phosphorylation sites were located at serine 31, threonine 8 and tyrosine 5, but the consequences of PBF hyper phosphorylation are unknown.

#### Nuclear localization signal

Due that PBF was initially identified as a papillomavirus transcription factor; we searched for a nuclear localization signal (NLS) in PBF, using several servers, the cNLS Mapper, NucPred, and PSORT II. All of them predicted one NLS within the sequence of PBF; probably a monopartite signal located at the amino acid residues 267–277. The NLS was found within an ordered region of PBF, near the zinc finger domain (Table 2). Even more, PSORT II predicted PBF localization mainly in the cellular nucleus (69.6%), and 21.7% in mitochondria. Data in agreement with the PBF localization reported by Boeckle et al., 2002 [2].

### Intermolecular interactions involving PBF

Due that PBF has demonstrated its capacity to interact with two cellular proteins, 14-3-3β and Scythe/BAT3, which are involved in important cellular processes such as apoptosis, signaling and cell growth control, we used *in silico* analysis to identify other important interactions. For that, we determined the probable PBF domains using the MAMMOTH analysis (program included in Robbeta service). Four PBF domains were predicted: the first one, located at residues 1–125; the second at 126–215, the third at 216–301 and the fourth at 302–483 (Table 2). The first and second domains probably have relevant function, but up to now there are not any experimental evidences to confirm that.

The transcriptional function of PBF is probably located in the third domain, which by the analysis of level of disorder, it was ordered, and contained the putative zinc finger structure type III, at amino acid residues 279–302 [1] (Figure 5B). This region was predicted also by the secondary and tertiary structure of the PBF, as a classical zinc finger structure; with an alpha helix and a beta sheet, flanked by flexible and disordered regions. This structure might enable PBF to catch the target DNA molecule to carry out its transcription activity [26].

The fourth domain located at residues 302–483, had a high disorder level, and also, it contained 18 phosphorylation sites. This domain was previously identified as the PBF binding site to the 14-3-3β protein [5].

**Figure 7:**
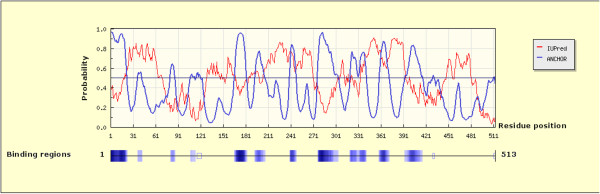
**Probable PBF binding sites using the ANCHOR server.** The graph shows the probable binding sites along the amino acids sequence of PBF to cellular factors (blue line). This server simultaneously shows in red color the disordered regions of PBF. The lower scale shows the probability of PBF interaction, dark blue color is used for region with high probability, and light blue indicates the lowest probability of binding.

**Figure 8:**
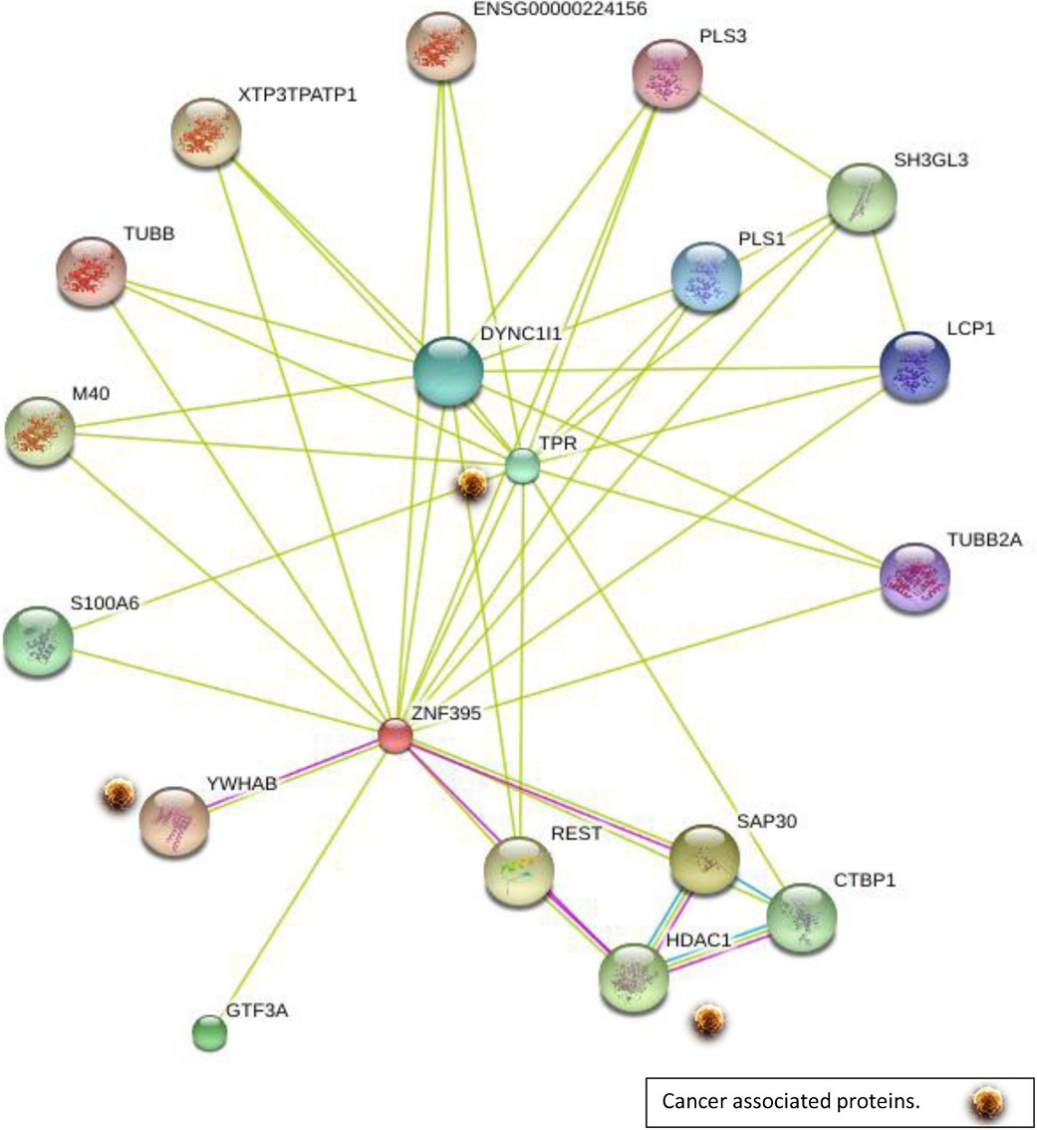
**Probable PBF interaction with different cellular proteins, using the network STRING server.** This figure shows the probable cellular factors capable to bind to PBF protein, but it does not indicate the position of the biding site. Among the identified factors, three cellular factors TPR, HDAC1 and YWHAB were detected and these cellular proteins are known to be associated to several cancers.

To find out other probable PBF interactions, we used the ANCHOR server, finding a total of 14 probable binding sites for cellular factors, 7 of them with high probability to bind PBF, at positions 1 to 22, 166 to 182, 193 to 206, 277 to 304, 320 to 341, 359 to 372 and 393 to 416 (Figure 7). The ANCHOR analysis was complemented with the STRING server. This server has the option of displaying up to 50 interactions; but we focused only on interactions with proteins related to neoplasias (Figure 8). Among these proteins we identified the 14-3-3β, also known as YWHAB, which has been previously demonstrated its binding capacity to PBF [27]. Besides that, other probable PBF interactions were detected, such as the HDAC1 (histone deacetylase) and the TPR (Translocated promoter region protein, nuclear basket protein), proteins which are known to be deregulated in different types of cancer [28, 29]. HDACs were thought to be recruited predominantly by transcriptional repressors to facilitate local histone deacetylation and transcriptional repression; but more recently genome-wide assays have mapped HDAC1/2 and their associated proteins to transcriptionally active loci, whereby their repressive functions are subtly exerted to balance transcriptional activation and repression. Therefore an interaction with overexpressed PBF could lead to keep up several transcriptional sites which otherwise should be repressed by deacetylation with HDACs [30].

The TPR protein is a component of the nuclear pore complex (NPC), a complex required for nucleus-cytoplasmic transport of proteins. TPR interaction with PBF, may be another strategy used by PBF to shuttle from cytoplasm to nucleus [29].

Other ligand for PBF is the DNA, and it has been demonstrated that PBF recognizes the sequence CCGG within the papillomavirus promoter [2]. Recently, it has been reported that union between DNA and PBF under hypoxic conditions could be involved in cancer progression, due to activation of several genes; such as the hypoxia response elements, specifically by overexpression of the hypoxia-inducible transcription factor-1α (HIF-1α), which induces expression of pro-inflammatory proteins, angiogenesis and cancer progression [31]. Table 2 shows the functional regions detected in the PBF amino acids sequence and its interacting or binding capacity to several key cellular proteins.

## Conclusions

*In silico* analysis of PBF physicochemical properties supported that PBF can be considered as an intrinsically disordered protein; which besides its known interactions with the cellular 14-3-3β and Scythe/BAT3 proteins showed probable interactions with other cellular factors such as HDACs and TPR. All these interactions together with the original role of PBF as a transcription factor, suggest that PBF potentially might be able to participate in osteosarcoma genesis by deregulation of the apoptosis mechanisms and cellular transcription control. Identification of these specific interactions is important to understand the carcinogenesis process which might allow the identification of new targets for diagnosis and treatments.

### Computational methods

The amino acids sequence for PBF protein was obtained from the NCBI database (http://www.ncbi.nlm.nih.gov/) using the access number **Q9H8N7.2.**

#### Disorder prediction

To analyze if the PBF protein might be an intrinsically disordered protein, we used the MetaDisorder web service (http://iimcb.genesilico.pl/metadisorder/). This server generates a consensus sequence based on the results of 13 web services [32]. It includes: DisEMBL, which predicts classic loops (DSSP), flexible loops with high B-factors, missing coordinates in X-ray structures, regions of low-complexity and prone to aggregation. DISOPRED2 predicts residues with missing coordinates, using neural networks. GlobPlot method is based on several hydrophobicity scales to predict regions of missing coordinates and loops with high B-factors. iPDA, which incorporates information about sequence conservation, predicts secondary structure, sequence complexity and hydrophobic clusters. IUPred estimates pairwise interaction energies using a statistical potential. Pdisorder server uses neural network, linear discriminant function and acute smoothing procedure for recognition of disordered and ordered regions in proteins. Poodle-s for short disorder detection (uses PSSMs generated by PSI-BLAST). Poodle-l predicts long disorder. PrDOS predicts missing coordinates in 3D structure. Spritz predicts long and short disorder, using secondary structures. RONN predicts missing coordinates.

#### Physicochemical analysis

Physicochemical properties of PBF, such as amino acids composition, were determined using ProtParam (http://web.expasy.org/protparam/) [33].

Intrinsically disordered proteins, also share other characteristics such as high flexibility level, abundance of hydrophilic amino acids and charge regions; for that reason we analyzed these properties for PBF [7]. Hydrophobicity was determined using ProtScale (http://web.expasy.org/protscale/), with Kyte and Doolitle scale, which is based on experimental data for each amino acid. Average flexibility was determined with ProtScale available at ExPasy (http://web.expasy.org/protscale/). Net charge was determined using EMBOSS (http://emboss.sourceforge.net/apps/cvs/emboss/apps/charge.html).

#### Nuclear localization signal (NLS) prediction

For NLS prediction we used three servers: 1) cNLS Mapper server (http://nls-mapper.iab.keio.ac.jp/cgi-bin/NLS_Mapper_form.cgi). It predicts nuclear localization signals (NLSs) specific to the importin αβ pathway. The profiles are generated by amino acids analysis for each NLS class in budding yeast [34]. 2) NucPred (http://www.sbc.su.se/~maccallr/nucpred/). It analyzes a eukaryotic protein sequence and predicts if the protein spends at least some time in the nucleus or spends no time in the nucleus. NucPred is an ensemble of 100 sequence based predictors [35]. 3) PSORT WWW Server (http://psort.hgc.jp/), this program predicts the subcellular localization sites of proteins from their amino acids sequences [36].

#### PBF crystallization probability

It is known that proteins with disordered regions have low propensity to be crystallized [13], for that reason we analyzed the probability of PFB to be crystallized using the XtalPred server (http://ffas.burnham.org/XtalPred/help.html) [37, 38]. This method identifies several protein features that correlate strongly with successful protein production and crystallization and combine them into a single score that assesses "crystallization feasibility”. Such features include protein length, molecular mass, gravy index, instability index, extinction coefficient, and isoelectric point.

#### 2D and 3D structure prediction

To determine the probable 2D structure for the PBF, we used PSIPRED (http://bioinf.cs.ucl.ac.uk/psipred/) [39]. PSIPRED is a simple and accurate secondary structure prediction method, incorporating two feed-forward neural networks which perform an analysis on output obtained from PSI-BLAST (Position Specific Iterated -BLAST) [40].

The 3D prediction was made with robetta (http://robetta.bakerlab.org/). This server uses the first fully automated structure prediction procedure that produces a model for an entire protein sequence in the presence or absence of sequence homology to protein(s) of known structure [41].

### Quality of the probable structures

The stereochemistry quality of the 3D models was measured with Procheck server UCLA MBI—SAVES (http://services.mbi.ucla.edu/SAVES/).

For the 3D structure refinement we used Yasara server (http://www.yasara.org/minimizationserver.htm).

#### Phosphorylation sites

Other important post-transcriptional modification in proteins is the phosphorylation, and it is a very important regulation way for IDPs, so we analyze probable phosphorylation sites using the **NetPhos 2.0 Server (**http://www.cbs.dtu.dk/services/NetPhos/). This tool produces neural network predictions for serine, threonine and tyrosine phosphorylation sites in eukaryotic proteins [42].

#### Partner protein binding sites

It is known that IDPs have many sites for binding several proteins or receptors. For this reason we used the ANCHOR server. This server compares the target protein with known globular proteins and considerers three criterions to predict the binding sites. The first criterion ensures that a given residue belongs to a long disordered region, and filters out globular domains. The second corresponds to the isolated state and it ensures that a residue is not able to form enough favorable contacts with its own local sequential neighbors to fold; otherwise it would be prone to adopt a well-defined structure on its own. The third tests the feasibility of a given residue to form enough favorable interactions with globular proteins upon binding (http://anchor.enzim.hu/) [43, 44].

#### Interaction with other proteins

To confirm the data obtained with the ANCHOR server, we used the STRING server (http://string-db.org/), which uses a database of known protein interactions, the interactions include direct (physical) and indirect (functional) associations derived from four sources, genomics, experiments, co-expression and prior knowledge [45].

## Abbreviations

PBF: Papillomavirus binding factor
IDP: Intrinsically disordered proteins
14-3-3β protein or YWHAB: Tyrosine 3-monooxygenase/tryptophan 5-monooxygenase activation protein, beta
Scythe/BAT3: Large proline-rich protein
HDAC: Histone deacetylase
TPR: Translocated promoter region protein or nuclear basket protein
ZNF593: Zinc finger protein
HIF-1α: Hypoxia-inducible transcription factor-1α.
